# Giant Desmoid Tumor of the Anterior Abdominal Wall in a Young Female: A Case Report

**DOI:** 10.1155/2013/780862

**Published:** 2013-04-21

**Authors:** Mahim Koshariya, Samir Shukla, Zuber Khan, Vaibhav Vikas, Avinash Pratap Singh, Puspendra Baghel, Varun Pendro, Vishal Kirti Jain, Shrikant Jagdish Jai, Sanjeev Kumar, M. C. Songra

**Affiliations:** Department of Surgery, Gandhi Medical College & Associated Hamidia Hospital, Bhopal 462001, India

## Abstract

Desmoid tumors (also called desmoids fibromatosis) are rare slow growing benign and musculoaponeurotic tumors. Although these tumors have a propensity to invade surrounding tissues, they are not malignant. These tumors are associated with women of fertile age, especially during and after pregnancy. We report a young female patient with a giant desmoid tumor of the anterior abdominal wall who underwent primary resection. The patient had no history of an earlier abdominal surgery. Preoperative evaluation included abdominal ultrasound, computed tomography, and magnetic resonance imaging. The histology revealed a desmoid tumor. Primary surgical resection with immediate reconstruction of abdominal defect is the best management of this rarity. To the best of our knowledge and PubMed search, this is the first case ever reported in the medical literature of such a giant desmoid tumor arising from anterior abdominal wall weighing 6.5 kg treated surgically with successful outcome.

## 1. Introduction

Desmoid tumors (also called desmoids fibromatosis) are rare slow growing benign fibrous tumors without any metastatic potential but a strong tendency to invade locally and to recur. The term desmoids was coined by Muller in 1838 and is derived from the Greek word *desmos,* which means tendon like. These tumors often appear as infiltrative, usually well differentiated and locally aggressive in nature and also known as deep fibromatoses. Desmoid tumors may be classified as extra-abdominal, abdominal wall, or intra-abdominal (the last is more common in patients with familial adenomatous polyposis (FAP)). It is thought that the lesions may develop in relation to estrogen levels or trauma/operations. They constitute 3% of all soft tissue tumors and 0.03% of all neoplasms [1]. These tumors are associated with women of fertile age, especially during and after pregnancy. The most common site of occurrence of desmoids is the anterior abdominal wall, with an incidence of 50% [1–8]. 

## 2. Case Report

A 25-year-old female patient presented to surgery department with a painless mass in the lower abdomen for 15 months which was gradually increasing in size. The patient had no history of previous abdominal surgery or any abdominal trauma. 

**Figure 1:**
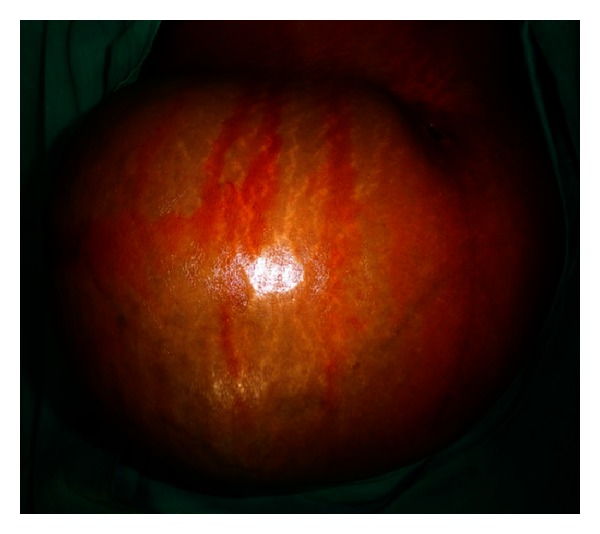
Preoperative image showing huge lump located in the anterior abdominal wall.

On examination, a single lower abdominal mass involving right lumbar, umbilical, hypogastric, and both iliac regions and extending vertically 6 cm below xiphisternum to pubic symphysis and transversely from right anterior axillary line to left anterior axillary line (Figure 1). 

Lump was fixed to the anterior abdominal wall, nontender, globular in shape of size 25 × 15 × 20 cm with smooth surface, and firm in consistency. 

Ultrasonography demonstrated a large solid heterogeneous hypoechoic mass showing internal vascularity, few anechoic areas of necrosis, seen in pelvic cavity extending to right lumbar region displacing the bowel loops superiorly.

**Figure 2:**
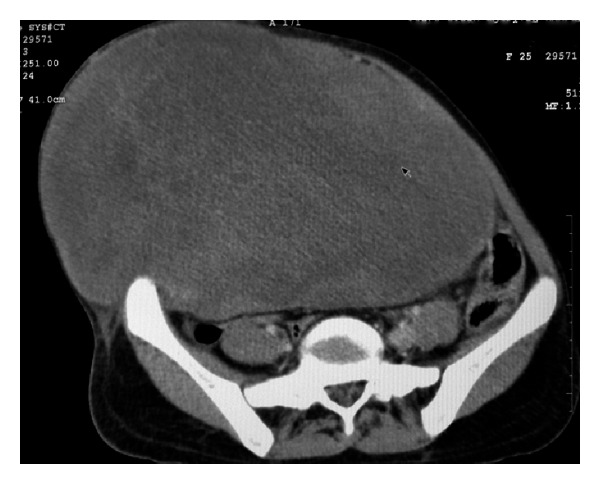
CT scan showing mass arising from anterior abdominal musculature.

CT scan revealed a neoplastic anterior abdominal muscle mass arising from right anterolateral abdominal wall muscles in right lumbar, right iliac fossa, crossing midline extending to involve contralateral anterolateral abdominal wall muscles, anteriorly causing stretching of subcutaneous tissue. Intra-abdominal extension of mass reaching up to anterior surfaces of lower lumbar vertebrae mildly compresses aorta and IVC and displaces underlying bowel loops and bladder. Differentials include desmoid tumor and rhabdomyoma. No evidence of lymphadenopathy was detected. No evidence of free fluid was seen in pelvic and peritoneal cavity (Figure 2). 

**Figure 3:**
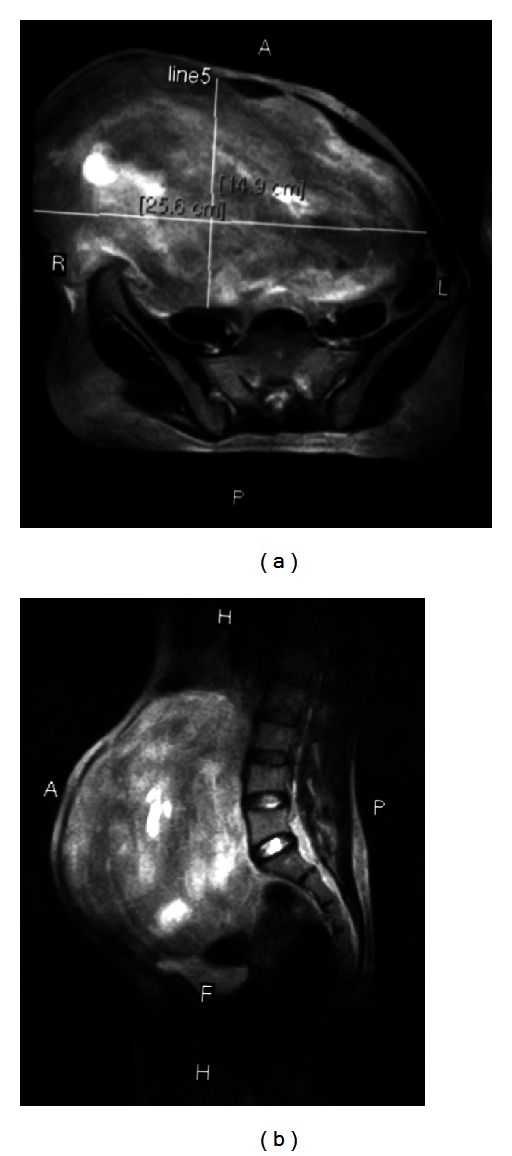
MRI images.

**Figure 4:**
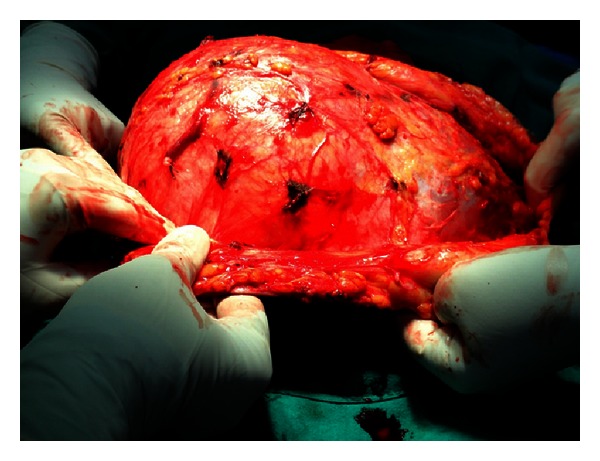
Intraoperative image.

**Figure 5:**
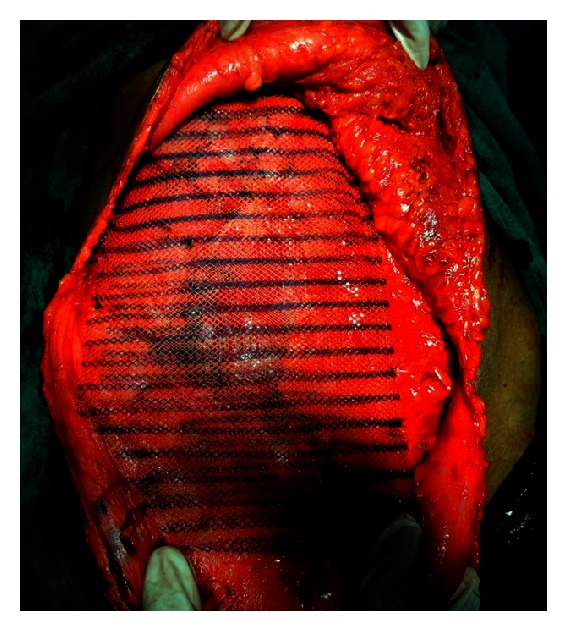
Image showing reconstruction of defect with mesh.

**Figure 6:**
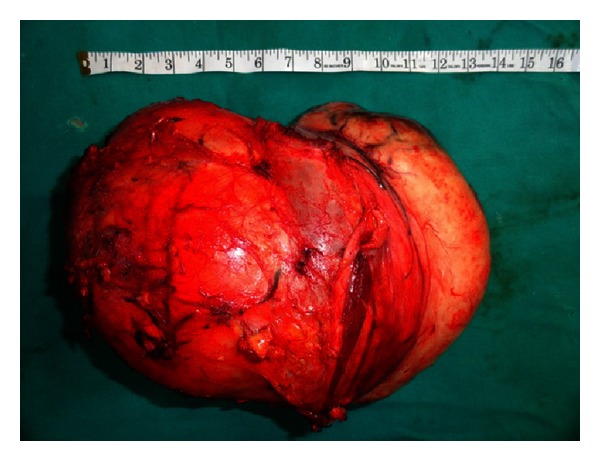
Showing excised specimen.

**Figure 7:**
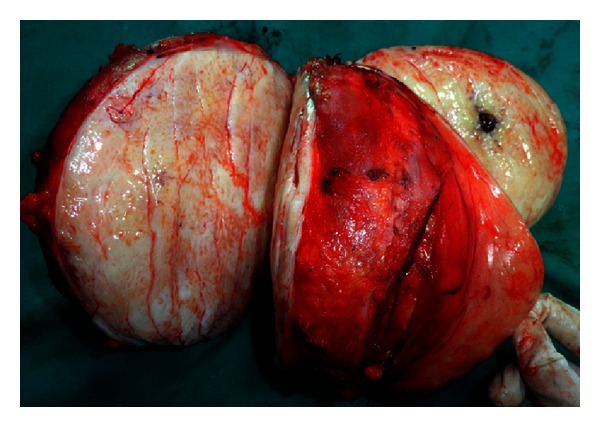
Cut section of the tumor.

MRI abdomen and pelvis (Figures 3(a) and 3(b)) revealed a large mass, measuring 20.6 (SI) × 24.6 (RL) × 14.6 (AP) cm arising from the right anterior abdominal wall musculatures having large component superficial to the muscle plane and similar large component deep/abdominal cavity to the muscles plane with central necrotic component. Fat plane to the surrounding organs appears normal. No enlarged lymph node or ascites were seen. No focal lesion was noted in bone or liver. Described MRI imaging features were compatible with neoplastic anterior abdominal wall origin mass and considered for desmoid tumor. After preoperative workup, patient was planned for operation, and complete excision of the tumor (Figure 4) with wide surgical margins along with the anterior abdominal wall down to the peritoneum was performed, resulting in a large wall defect of about 18 × 20 cm which was repaired after mobilization and release of rectus abdominis and reconstructed with polypropylene + Monocryl mesh (Figure 5). Skin was closed after keeping vacuum suction drain beneath the subcutaneous space. Excised mass was heavy weighing 6.5 kg (Figure 6), and on cut section, it was gritty texture and glistening white in colour (Figure 7). 

The postoperative course was uneventful, and patient was discharged on the 9th postoperative day. Histopathological reports were consistent with desmoid tumor with negative surgical margins. At 8 months of followup, patient did not have any recurrence or incisional hernia. 

## 3. Discussion

Desmoid tumors are cytologically benign fibrous neoplasm originating from the musculoaponeurotic structures throughout the body. 

Desmoid tumors are benign deep aggressive fibromatoses, originating from fascia and muscle aponeurosis with an infiltrating growth [2]. Approximately, 3.7 new cases occur per one million persons per year and often associated with female gender, FAP [4], estrogen therapy, and occasionally with surgical trauma (1 in 4 cases). Trauma especially operative trauma may contribute to the formation of desmoid tumors [5]. Estrogen may act as a growth factor. Endogenous estrogen levels have a close correlation with tumor growth factor rate. It has a higher prevalence in young women who experienced pregnancy [6] and rare in males. They may be extra-abdominal (the shoulder girdle, trunk, and lower extremities), intra-abdominal (in the abdominal wall, especially the rectus and internal oblique muscles with their fascial coverings, and mesentery or retroperitoneum), multiple familial, and as part of Gardner's syndrome. 

Abdominal desmoid tumor usually presents as a firm mass with ill-defined margins and no distinct capsule [8]. On cut surface, they are gritty, glistening white and trabeculated resembling scar tissue. Histologically, desmoid tumors consist of elongated fibroblasts and myofibroblasts [1–3, 7]. 

On ultrasonography, desmoid tumors appear as well-defined lesions with variable echogenicity. On CT, they may appear as homogeneous or heterogeneous and hypo-, iso-, or hyperintense compared with the attenuation of muscles [2, 6, 8, 9]. Characteristic MRI findings include poor margination, low-signal intensity on T1-weighted images and heterogeneity on T2-weighted images, and variable contrast enhancement [2, 5, 7]. MRI is superior to CT scanning in defining the pattern and the extent of involvement as well as in determining if recurrence has occurred after surgery, though both the modality CT and MRI aid in determining the extent of local invasion. 

Wide local excision with reconstruction of the defect is the treatment of choice. Peritoneum, intraperitoneal organs, or adjacent bony structures involved by tumor must be resected as well. Incomplete tumor removal or positive surgical margins may lead to local recurrence [1–6] (20% to 77% depending on the location, extent, and completeness of the initial resection). Abdominal wall desmoid tumors have a significantly lower recurrence rate (20% to 30%) and usually become evident within six months after excision. Radiotherapy, chemotherapy, and endocrine therapy are used in patients with inoperable tumors, local recurrences, or incompletely excised lesions [1, 2, 4, 5]. Metastatic disease has not been reported with desmoid tumor [2–4, 6–8]. Only few cases of malignant transformation in desmoid tumor are reported, and all were associated with local irradiation. Sulindac, indomethacin [10], and tamoxifen along with varieties of antineoplastic medications have been used to treat these patients with variable results. 

## 4. Conclusion

The history of painless abdominal mass, the age and sex of the patient, the location of the mass within the anterior abdominal wall, and the imaging features make desmoid tumor a strong primary diagnostic consideration even if it is a rare entity. Aggressive, wide surgical resection with negative margin is the best surgical option. Complete surgical excision of desmoid tumors is the most effective method of cure, sometimes necessitating removal of most of an involved anterior abdominal wall in such a giant desmoid tumor with immediate repair of resultant huge defect and reconstruction using prosthetic mesh for better functional results.
